# Syphilis response policies and their assessments: A scoping review

**DOI:** 10.3389/fpubh.2022.1002245

**Published:** 2022-09-16

**Authors:** Milena C. D. Almeida, António M. R. Cordeiro, Aliete Cunha-Oliveira, Daniele M. S. Barros, Diana G. S. M. Santos, Thaísa S. Lima, Ricardo A. M. Valentim

**Affiliations:** ^1^Centre for Interdisciplinary Studies, University of Coimbra, Coimbra, Portugal; ^2^Laboratory of Technological Innovation in Health (LAIS), Federal University of Rio Grande do Norte (UFRN), Natal, Brazil; ^3^Health Sciences Research Unit: Nursing (UICISA:E), School of Nursing of Coimbra (ESEnfC), Coimbra, Portugal; ^4^Coimbra Hospital, University Center, Coimbra, Portugal; ^5^Brazilian Ministry of Health, Brasília, Brazil; ^6^Department of Biomedical Engineering, Federal University of Rio Grande do Norte (UFRN), Natal, Brazil

**Keywords:** syphilis, evaluation, health policy, healthcare policy, public health policy

## Abstract

Syphilis is one of the most common sexually transmitted infections (STIs) worldwide and has shown a rising trend in recent years, according to a report published by the World Health Organization (WHO) in 2021. Given this problem, the present study aims to develop a scoping review of what has been done in the world after the publication of the global strategy for the elimination of STIs, with a specific focus on syphilis. Thus, we searched for papers on health policies in response to syphilis in Pubmed, Scopus, ScienceDirect, and EBSCO by CINAHL, as well as in official documents from international health organizations. The period from January 1, 2016, to August 14, 2022 was considered. Our search returned 880 papers addressing “Syphilis,” “Health Policy,” and “Health Policies” combined. Twenty-three papers fulfilled the inclusion and exclusion criteria according to two research questions set out for this scoping review. Our findings suggest that Brazil and Peru presented the greatest adequacy of the strategies provided by WHO in 2016 and the Pan American Health Organization (PAHO) in 2017, aiming tothe goals set out in the UN's 2030 Agenda for sustainable development. Among the studies found, six countries (Cuba, Thailand, Belarus, Armenia, Moldova, and Puerto Rico) reported the elimination of mother-to-child transmission (MTCT) of syphilis, but the most recent data are from 2016. Furthermore, it is essential to mention that no country has been found that has presented a comprehensive response to syphilis, noting the control or elimination of the disease in all key populations. Thus, it is necessary to constantly monitor national policies based on in-depth studies on the quality of the response, the challenges, and the national, regional, and global perspectives for the control of the disease until 2030, the year in which the SDGs will be reviewed.

**Systematic review registration:**

https://osf.io/x9er5/?view_only=0cc0062222ec45dcb2f4d41484d285b6, identifier: 10.17605/OSF.IO/X9ER5.

## Introduction

According to LaFond and Lukehart (1), syphilis is a sexually transmitted infection (STI) caused by *Treponema pallidum* subsp. *pallidum* (*T. pallidum*) and can occur in different stages. Syphilis is one of the most common STIs worldwide, with almost 6 million new cases yearly. If an infected pregnant woman does not receive adequate early treatment, she can transmit the infection to the fetus (MTCT), which can result in low-birth-weight newborns, premature birth, miscarriage, stillbirth, and early and late maternal and pediatric clinical conditions. In 2016, there were more than half a million cases of syphilis (about 661,000), which resulted in more than 200,000 stillbirths and neonatal deaths (2).

Despite syphilis being treatable with inexpensive and effective antibiotic therapy, it is a condition that remains prevalent as a public health threat, especially in high-income countries (3).

In 2016, based on the analysis of global data on the epidemiological behavior of STIs through the years 2006–2015 and on a careful assessment of the implications of syphilis on advancements in universal health coverage, the World Health Organization (WHO) launched a Framework for the prevention and control of STIs in the period from 2016 to 2021, containing the “Strategic guidelines for the elimination of sexually transmitted infections” (4). In it, WHO set as macro-objectives the reduction of 90% in the incidence of *T. pallidum* and 50 cases of congenital syphilis (CS) per 100,000 live births in 80% of countries.

Specifically for CS, WHO published in 2008 the global initiative to eliminate MTCT (5), and in 2017, the Pan American Health Organization (PAHO) presented the Framework entitled “Elimination of Mother-to-Child Transmission of HIV, Syphilis, Hepatitis B and Chagas” (6). The latter and the 2016 strategy were developed within the framework of achieving the global goals set out in the Sustainable Development Agenda for 2030.

Despite public health efforts, recent information from the Global Progress Report on HIV (7), Viral Hepatitis, and STIs reports an incidence of 7.1 million (3.8–10.3 million) new infections by *T. pallidum* between 2016 and 2020. Moreover, syphilis prevalence among people aged 15–49 years was 0.6% (0.5%−0.7%) in the same time period. Such data underscores the need for maintaining actions to tackle the disease across countries for the goals set forth in UN's 2030 Agenda to be promptly reached.

Considering the syphilis issue at a global level, this study aims to perform an scoping review that sought to answer two research questions, as follows: (1) How did the syphilis response was implemented in countries after the publication of international strategies related to STIs and to congenital syphilis from 2016? (2) Did the response to syphilis in these countries be assessed and what methods were used to assess these interventions? For this, the acronym PCC (Population, Concept, and Context) was used: Population: Any population; Concept: Health policies to respond to syphilis; Context: World.

In this context, this article aims to identify and analyze studies and research dealing with health policies for the response to syphilis and their descriptions and evaluations. It intends to present possible gaps in the literature concerning the WHO recommendations and contribute to future studies considering the global goal of eliminating syphilis by 2030 and strengthening the countries strategies.

### WHO framework for the global control of STIs, including syphilis

**Figure 1:**
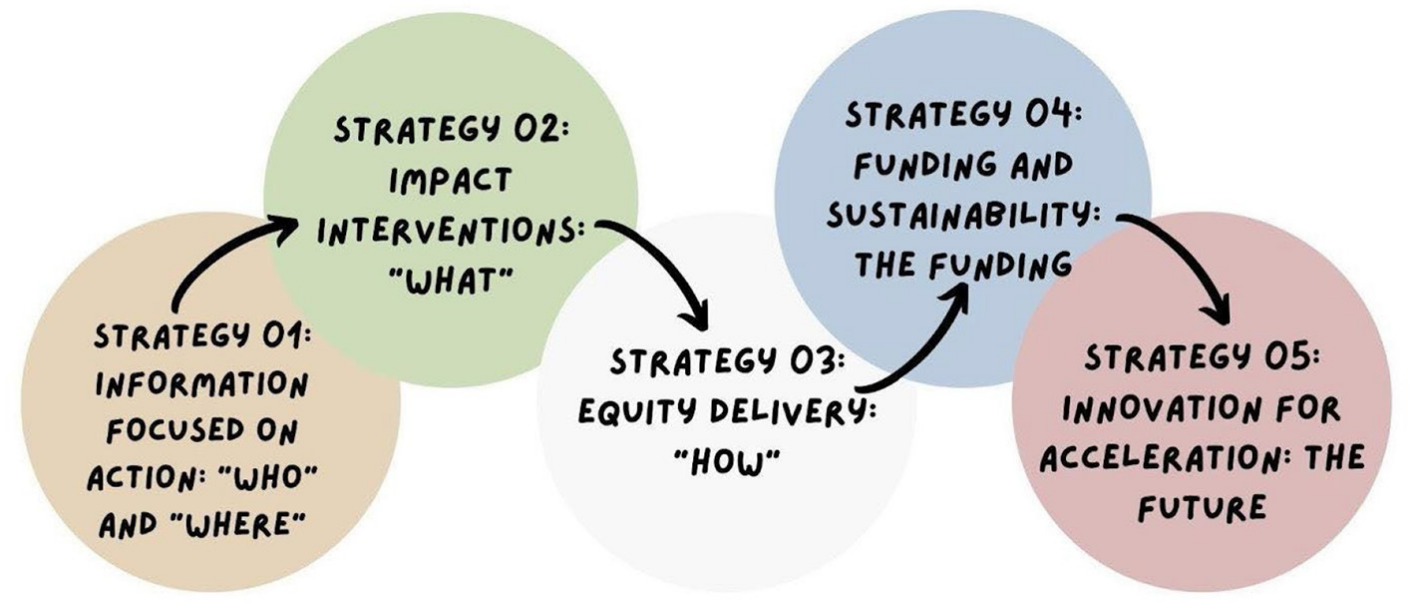
Strategic directions for eliminating sexually transmitted infections. Source: Adapted from WHO (2016).

To continue with the global control of STIs as well as to provide universal health coverage with the continuity of services related to the problem, Figure 1 shows the strategic directions for achieving the plan proposed by the WHO (2016) (4).

Figure 1 represents five strategies based on the following guidelines (4):

1. Strategy 01 – Action-focused information: Addresses the need to understand the STI epidemic, demarcating its response based on law, political commitment, national planning, resource mobilization, allocation, implementation, and the improvement program.
2. Strategy 02 – Interventions for impact: Handles the first dimension of universal health coverage, describing the essential and high-impact interventions that must be implemented along the STI care line. Health systems must consider that to achieve national and global goals.
3. Strategy 03 – Delivery for equity: Manages the second dimension of universal health coverage, presenting the best methods and approaches to be considered by the STI care line for different populations and in other locations to achieve equity, maximize the impact and guarantee the quality of services. It includes approaches focused on human rights, gender equality, and barriers that undermine the equitable access of different populations to services, environments, and places.
4. Strategy 04 – Financing and sustainability: Approaches the third dimension of universal health coverage, identifying sustainable and innovative solutions through financing models for the response to STIs, as well as approaches to reduce costs, with a focus on scaling up access to health services.
5. Strategy 05 – Innovation for acceleration: Identify areas where there are significant gaps in knowledge and technologies. Therefore, innovation is needed to change the trajectory of response to STIs.

### PAHO/WHO framework for the elimination of mother-to-child transmission of HIV, Syphilis, Hepatitis B, and Chagas

**Figure 2:**
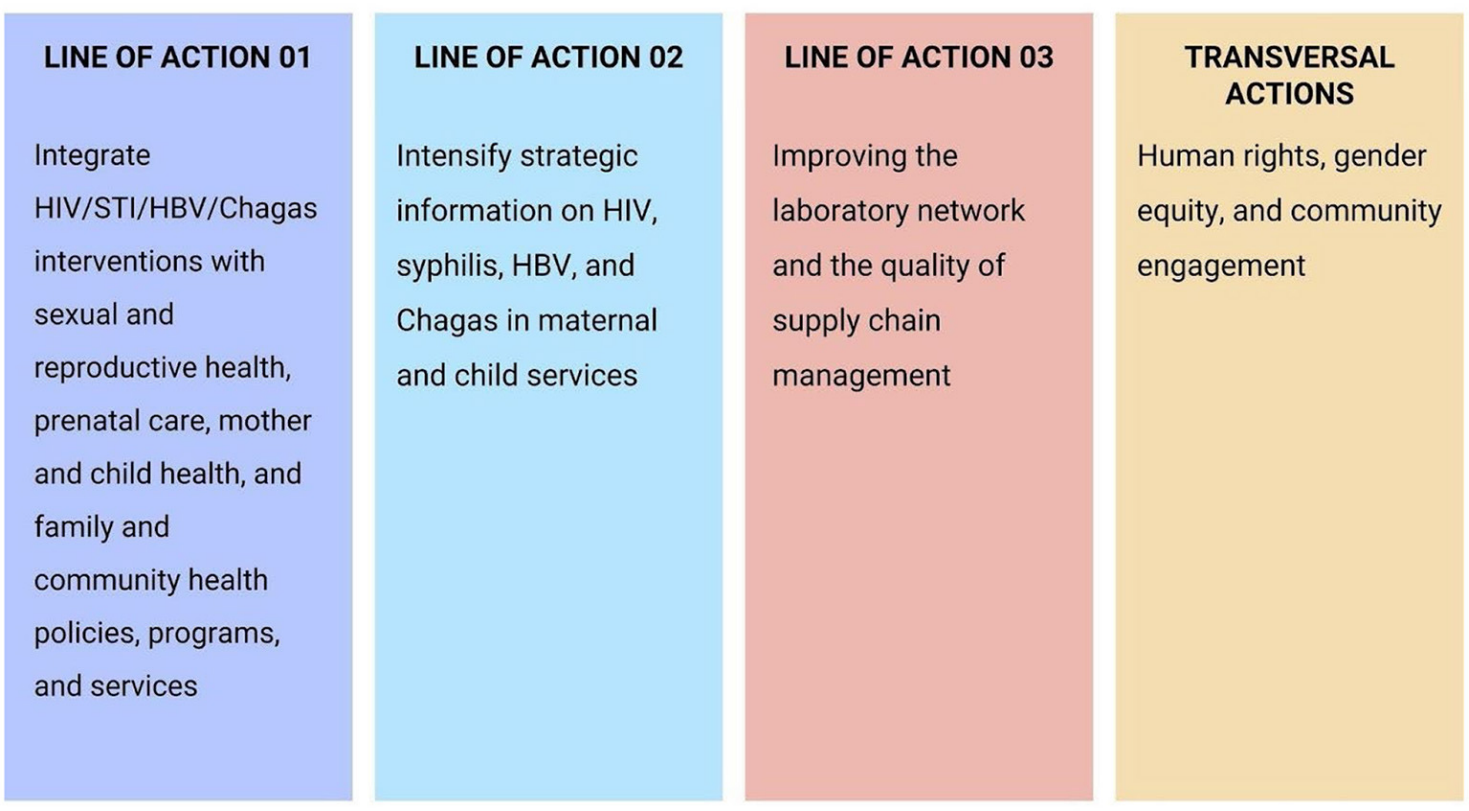
Framework for the Elimination of MTCT of HIV, Syphilis, Hepatitis B, and Chagas. Source: Adapted from PAHO (2017).

Since 2010, the member states of the Pan American Health Organization have committed to the elimination of mother-to-child transmission of HIV and syphilis in the Region, as a result, targets for 2015 were established through Resolution CD50.R12. In 2016, these commitments were renewed and expanded through the Plan of Action for the Prevention and Control of HIV and Sexually Transmitted Infections (2016–2021) to eliminate HIV and STIs in the Americas (6), as shown in Figure 2.

Figure 2 represents a conceptual framework after the lessons learned in the Dual Elimination process of HIV and syphilis in 2015 in Cuba and 2016 by six countries and territories (Thailand, Belarus, Anguilla, Montserrat, Armenia, and the Republic of Moldova) (6).

Thus, when expanding and renewing the agreement to eliminate mother-to-child transmission of HIV and STIs, the lines of action of the PAHO Framework perform the following steps (6):

1. Line of action 01: (a) evidence-based, (b) consistent with national/regional health priorities as outlined in national health plans and program-specific strategies, and (c) targeted to address gaps in health coverage interventions in all care as well as improvement in delivering quality service.
2. Line of action 02: Highlights the need to strengthen monitoring and evaluation and surveillance systems in all program areas, including primary prevention, antenatal care, diagnosis and treatment, linkage and retention to care and follow-up.
3. Line of action 03: National system of reference laboratories that guarantees high-quality services and supports lower-level laboratories is critical to address the elimination strategy.
4. Cutting actions: The integration of human rights implies the guarantee of freedom of choice and protection of autonomy, confidentiality, and informed consent in an equitable manner for all; gender equality is particularly relevant in the mother-to-child context of HIV and syphilis transmission, as gender norms and practices can significantly shape women's enjoyment of sexual and reproductive health and rights. This engagement must be multidimensional and must include the process of policy formulation, program development and implementation, advocacy, and service delivery.

Figures 1, 2 represent the main elements to be considered by countries to prepare their national responses and consequently achieve the objectives proposed by official WHO documents, in addition to the SDG 2030 Agenda.

## Methods

### Research strategies

This Scoping Review was carried out by reviewing multiple databases: Pubmed, Scopus, ScienceDirect and EBSCO by CINAHL, as well as official documents from international health organizations, focusing on syphilis response health policies. Considering publications from 2016 onwards, as this is the year of publication of the WHO's global strategy to eliminate sexually transmitted infections. The searches were operational until August 14, 2022. Thus, the selection of studies used the Joanna Briggs Institute (8) as methodology. In this review, the languages searched were English, Portuguese and Spanish.

A more global approach in the search terms “Syphilis,” “Health Policy,” and “Health Policies” was adopted to find a more significant number of publications that dealt with the topic and, in the screening process, research that described the response strategies to syphilis with assessment methods. This review was registered with Open Science (OSF) (9) through the link https://osf.io/x9er5/?view_only=0cc0062222ec45dcb2f4d41484d285b6.

### Inclusion and exclusion criteria

For the inclusion and exclusion of studies, criteria were structured to guide the selection of research by peer reviewers. Thus, the inclusion criteria were:

1. Articles published from January 1, 2016 to August 14, 2022, discuss health strategies and policies for the syphilis response. The period after 2016 refers to the WHO's publication regarding the global strategy to approach and eliminate Sexually Transmitted Infections.
2. Articles published in English, Spanish and Portuguese.

Regarding the exclusion criteria, the following were considered:

1. Articles unavailable, even after contacting the authors.
2. Clinical protocols for patients with syphilis.
3. Articles that do not discuss implemented syphilis response strategies and policies.
4. The exclusion of abstracts and papers published in congresses, news items, consulting documents, or documents without results on syphilis response policies, books, book chapters, and opinion articles happened due to the heterogeneity of records to compare the results found.

### Study screening

The study selection process followed the JBI Manual through two independent reviewers and was based on the inclusion and exclusion criteria of the studies. In this way, we evaluated titles and abstracts of the documents. With the differences in the selection process, there was a discussion to decide with the third reviewer what would be included or excluded at all stages. The whole process was performed on the Rayyan Platform (10).

### Data extraction

Data were extracted from a complete reading of the articles and documents included in this review and based on: (1) the guide proposed by the World Health Organization in 2016, which establishes a vision, objectives, goals, and guiding principles as well as priority actions for the elimination of sexually transmitted infections (4); and (2) The Framework published by (6) to guide countries toward the elimination of mother-to-child transmission of HIV, Syphilis, Hepatitis B and Chagas.

In this sense, the article emphasized the implemented strategies related to the response to syphilis. Thus, the data were analyzed and stratified according to the logic mentioned above in how the adopting strategies worked to answer the research questions for this study.

## Results

**Figure 3:**
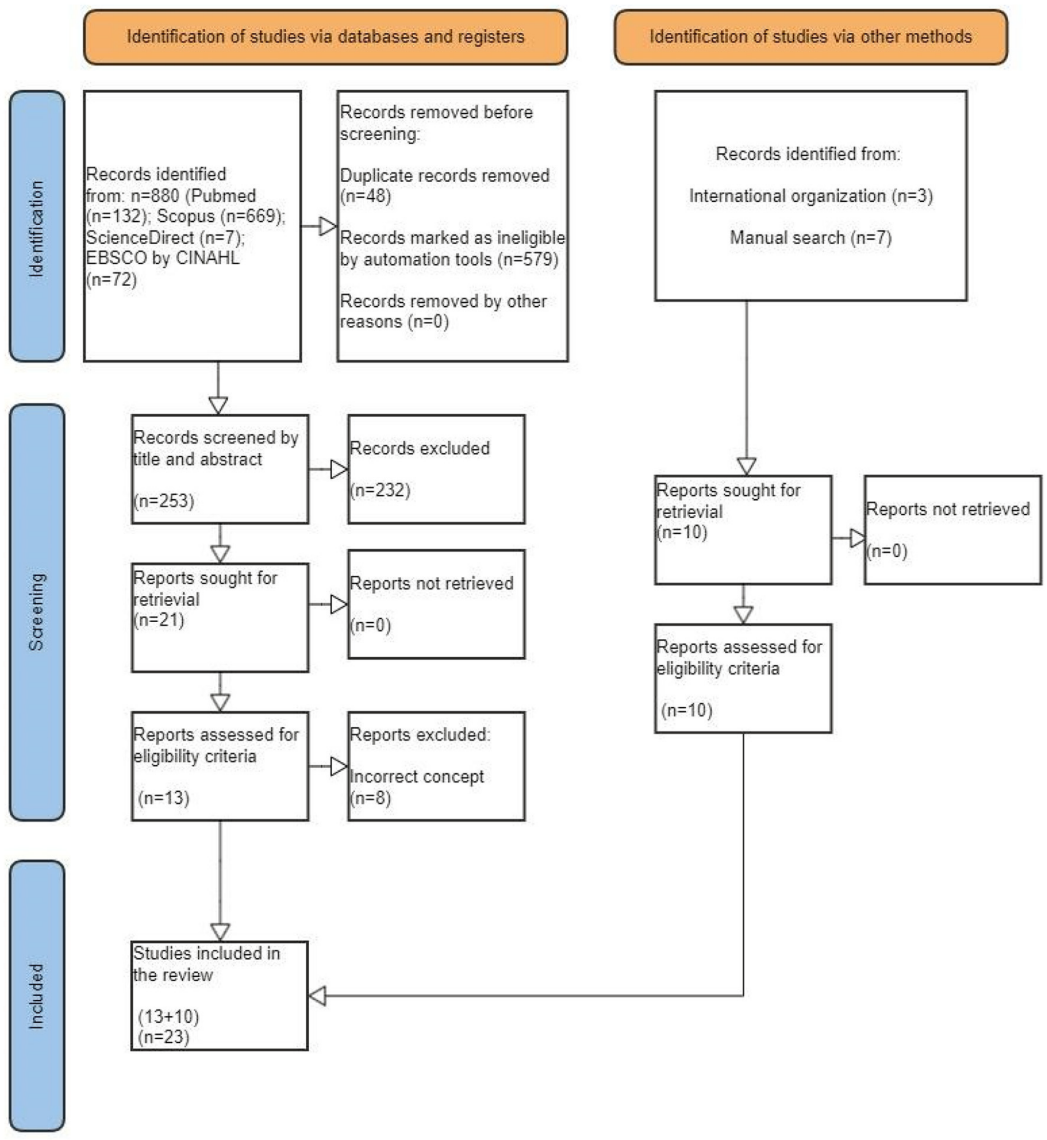
PRISMA ScR flow diagram: Scope review process for syphilis response.

The articles were extracted from Pubmed (*n* = 132), Scopus (*n* = 669), ScienceDirect (*n* = 7) and EBSCO by CINAHL (*n* = 72) databases, totaling 880 articles. Thus, two authors applied the eligibility criteria for inclusion. Thus, the evaluators used the Rayann platform for abstract and title screening. Then, they managed to remove 48 duplicated articles and developed a blind review. The reviewers eliminated (R1 = 214) and (R2 = 196). The third reviewer resolved the “conflicts” and the “maybes.” For the reading of this scoping review, 21 articles were considered. After full reading, 13 articles persisted. The manual search found (*n* = 7) articles and 03 official reports from international organizations, totaling 23 documents in this review. Figure 3 summarizes the process of these results.

The information stratification was carried out to understand what was accomplished in the fight against syphilis and whether actions undertaken have been monitored and evaluated according to implementation strategies provided in guide (4) and to eliminate CS (6).

Based on the recommendations listed by WHO in 2016 and PAHO 2017 as well as in Table 1, we verify that in the selected studies, there are relationships between the strategies proposed by the agencies and the policies implemented in the countries. Thus, in terms of results, the surveys were stratified between 2016 and 2017.

**Table 1:** Strategies implemented according to the WHO 2016 framework.

**Authors**	**Year**	**Strategic direction 1: Information for focused action - the “who” and “where”?**	**Strategic direction 2: Interventions for impact - the “what”?**	**Strategic direction 3: Delivering for equity - the “how”**	**Strategic direction 4: Financing for sustainability - The financing**	**Strategic direction 5: Innovation for acceleration - The future**
Taylor et al. (11)	2017	Cuba, Thailand, Belarus, Armenia, and Moldova: **Congenital syphilis**	Universal care access, routine antenatal screening, legal frameworks, primary care services.	No	No	No
Leichliter et al. (12)	2017	USA: **Pregnant women**	Prenatal syphilis screening.	No	No	No
Chemtob et al. (13)	2017	Israel: **Sexually active adolescents and adults**	High-Intensity Behavioral Counseling; condoms distribution and STI testing	No	No	No
Rodrigues and Domingues (14)	2017	Brazil: **Pregnant women**	No	No	No	No
WHO (15)	2017	Asia: Cambodia (**congenital syphilis**) and China (**pregnant women**)	Cambodia: Evaluation of HIV–syphilis dual testing; China: National STI control program strategy is to integrate syphilis control activities with existing HIV control programs	No	No	No
Akhtar and Rehman (16)	2018	Lusaka, Zambia: **Pregnant women**	Universal antenatal screening	No	No	No
Ong et al. (17)	2018	Worldwide: **Key population**	Syphilis testing in key populations	Syphilis testing programs: Focused on MSM	No	No
Leal et al. (18)	2018	Brazil: **Pregnant women and congenital syphilis**	Universalization of medical assistance	Guaranteed by the SUS, with reducing inequalities in access and coverage	Provided by the Rede Cegonha Program	No
Kroeger et al. (19)	2018	Caddo Parish, Louisiana, USA: **Congenital syphilis**	No	No	No	No
Andrade et al. (20)	2020	Northeast, Brazil: **Pregnant women**	VDRL exams - up to the 20th week of pregnancy and between the 28th and 36th week of pregnancy	No	No	No
Acharya et al. (21)	2020	Nepal: **General syphilis**	STIs counseling, diagnosis and treatment services	No	No	No
Liu et al. (22)	2020	California, USA: **Key population**	Expansion of screened populations for syphilis and targeted screening interventions	No	No	No
Kimball et al. (23)	2020	Florida, Louisiana and New York, USA: **Pregnant women and congenital syphilis**	Syphilis testing early in the third trimester	No	No	No
Herrero et al. (24)	2020	Argentina: **Pregnant women and congenital syphilis**	Strategy to achieve elimination of the MTCT of HIV and congenital syphilis	No	No	No
Priamo et al. (25)	2020	Bahia, Brazil: **Pregnant women and congenital syphilis**	Planning actions to qualify prenatal care	No	No	No
Andrade et al. (26)	2020	Brazil: **Congenital syphilis**	Surveillance and health care, educommunication, governance, and syphilis research	Strengthening sexual health and reproductive health actions, especially in the context of primary care	Project “Syphilis No” - an integrated and collaborative response to syphilis that articulates health care points in an inter-federative relationship	Articulating social sectors and communities to strengthen a rapid response to syphilis
WHO (27)	2020	Ukraine: **Pregnant women**	Antenatal care, testing and treatment for HIV, and testing for syphilis	No	No	No
Sykes et al. (28)	2021	Arizona, USA: **Pregnant women**	Serologic testing for syphilis at 28 to 32 weeks of gestation, among “pregnant syphilis case(s)	No	No	No
Pinto et al. (29)	2021	Brazil: **General and congenital syphilis**	Implemented a network of Research and Intervention Supporters, to combat syphilis in priority municipalities; Campaign to fight the syphilis epidemic in the country	The role of the Supporters was to enhance action in priority municipalities and create a link between the Ministry of Health and local actors (municipal managers, health professionals, and the population)	Project “Syphilis No”: National Agenda for Strategic Actions to Reduce Syphilis in Brazil	Adaptive model for the discovery and temporal analysis of the health campaign reach. Were implemented into Hermes system, and it manages the complete data life cycle
PAHO (30)	2021	Peru: **Congenital syphilis and key population**	Use of rapid syphilis tests, rapid dual HIV/syphilis tests since 2017–2018, strengthening laboratory capacity and networks, strengthening STI case reporting, combination HIV/STI prevention	Decentralization of treatment to the first level of care	A National 2017–2021 Plan commits the country to eliminate congenital syphilis by 2021	Applied a compartmental dynamical model of adult syphilis transmission to examine possible future program scale-up scenarios and inform national STI control strategies and targets
Zorilla et al. (31)	2021	Puerto Rico: Pregnant women and congenital syphilis	Prenatal testing HIV/syphilis, allowed for the availability of treatment during pregnancy and entailed training of health professionals in obstetrical and pediatric hospitals	Safeguarding the commitment and investment in research, training, outreach, and women-centered care	Early screening policy	No
Du et al. 32]	2022	China: **Pregnant women and congenital syphilis**	Health care services were initially provided to all pregnant women with HIV/syphilis/hepatitis B who attended the national sentinel sites	Service network of women and children's health (WCH) has been gradually formed, which is composed of general hospitals, WCH institutions, and community-based healthcare services at multiple levels, province, district, county, township, and village	China launched a pilot project on the prevention of MTCT (PMTCT) in eight counties (cities, districts) of five provinces (regions) in 2002	No
Tang et al. (33)	2022	China: Congenital syphilis in Guangdong Province	Integrating the syphilis screening with health education, the standard treatment for pregnant women and the follow-up of infants of congenital syphilis patients, alongside supportive strategies from the government	The preventive and control measures	The National Program of Syphilis Control and Prevention 2010–2020	No

Source: Prepared by the authors (2022).

Regarding the analyzed studies, 19 papers (82.60%) have the approach to syphilis in pregnancy (SIP) and CS. In comparison, three articles (13.04%) focused on key populations (men who have sex with men, sex workers, people who use drugs, transgender, incarcerated people, and those experiencing homelessness). Moreover, two articles (8.69%) focused on syphilis in general terms, and lastly, only one study (4.34%) addressed policy oriented toward sexually active adolescents, considering this a vulnerable population.

Geographically, the Region of the Americas comprises most of the studies. Brazil and the United States have the largest number of publications that describe policies implemented to fight syphilis, which corresponds to six publications each (52.17%). Cuba, Argentina, Puerto Rico and Peru have only 01 publications each. The Asian continent registered six publications - Israel, Thailand, Cambodia and China (together in a WHO Report) and Nepal. In Europe, there are 02 studies, the first, integrates Belarus, Armenia, and Moldova into broader studies, and the second study is about Ukraine. Africa has recorded only 01 from Zambia.

All the 23 articles report “where” the syphilis response policy or intervention took place, which makes up 100% adherence to Strategy 01 of the WHO 2016 Framework. Regarding Strategy 02: Interventions for impact – “what,” 21 of them (91.30%) present elements about the policy or intervention that was implemented in the fight against syphilis. Only 02 articles (8.69%) do not explain how the procedure was conducted.

Regarding Strategy 03, eighth articles (34.78%) cite the equity attribute as fundamental for equal access to health services, especially concerning mother and child health. Seven studies (30.43%) have citations about funding for policies of syphilis reduction. They are concentrated in Latin America and Asia (Brazil, Peru and China). Three of the studies (13.04%) reported elements related to innovations for acceleration, which connects them with Strategy 5 of the WHO 2016 Framework.

Regarding the PAHO/WHO 2017 Framework lines of action, the distribution of results considered the 16 selected articles focused on SIP and CS. In Table 2, it is possible to verify how the revised texts adhered to the PAHO/WHO proposal.

**Table 2:** Lines of action implemented according to the PAHO/WHO 2017 framework.

**Authors**	**Year**	**Where and who**	**Line of action 1: Integrate HIV and STI interventions within sexual and reproductive health, antenatal care, maternal and child health, and family and community health policies, programs, and services**	**Line of action 2: Intensify strategic information on syphilis in maternal and child health services**	**Line of action 3: Improve the laboratory network and quality and supply chain management**	**Cross-cutting actions: Human rights, gender equality, and community engagement**
Taylor et al. (11)	2017	Cuba, Thailand, Belarus, Armenia and Moldova: **Congenital syphilis**	Yes	Yes	Yes	Yes
Leichliter et al. (12)	2017	USA: **Pregnant women**	Yes	No	No	No
Rodrigues and Domingues (14)	2017	Brazil: **Pregnant women**	Yes	No	No	No
WHO (15)	2017	Asia: Cambodia (**congenital syphilis**) and China (**pregnant women**)	Yes	No	No	No
Akhtar and Rehman (16)	2018	Lusaka, Zambia: **Pregnant women**	Yes	No	No	No
Leal et al. (18)	2018	Brazil: **Pregnant women and congenital syphilis**	Yes	No	No	Yes
Kroeger et al. (19)	2018	Caddo Parish, Louisiana, USA: **Congenital syphilis**	No	No	No	No
Andrade et al. (20)	2020	Northeast, Brazil: **Pregnant women**	Yes	No	No	No
Kimball et al. (23)	2020	Florida, Louisiana and New York, USA: **Pregnant women and congenital syphilis**	Yes	No	No	No
Herrero et al. (24)	2020	Argentina: **Pregnant women and congenital syphilis**	No	No	No	No
Priamo et al. (25)	2020	Bahia, Brazil: **Pregnant women and congenital syphilis**	Yes	Yes	No	No
Andrade et al. (26)	2020	Brazil: **Congenital syphilis**	Yes	Yes	Yes	Yes
WHO (27)	2020	Ukraine: **Pregnant women**	Yes	No	No	No
Sykes et al. (28)	2021	Arizona, USA: **Pregnant women**	No	No	Yes	No
Pinto et al. (29)	2021	Brazil: **General and congenital syphilis**	Yes	Yes	Yes	Yes
PAHO (30)	2021	Peru: **Congenital syphilis and key population**	Yes	Yes	Yes	Yes
Zorilla et al. (31)	2021	Puerto Rico: **Pregnant women and congenital syphilis**	Yes	No	No	No
Du et al. (32)	2022	China: **Pregnant women and congenital syphilis**	Yes	Yes	No	Yes
Tang et al. (33)	2022	China: Congenital syphilis in Guangdong Province	Yes	Yes	No	No

Source: Prepared by the authors (2022).

The distribution of the reviewed studies in b shows that only four of 19 studies (21.05%) include elements of the 4 lines of action of the 2017 Framework. The four studies were carried out in: South America; Europa and Asia. The latter was coordinated and published by PAHO itself (6).

As for the individual analysis by line of action, in Table 2, it is possible to verify that the line of action 1, which aims to integrate interventions to respond to HIV and STIs in sexual and reproductive health, prenatal care, health maternal and child health, as well as family and community health policies, programs and services, is the one with the highest adherence or correlation of studies and analyzes carried out in the countries, which makes a total of 18 articles (94.73%) of the 19 analyzed.

Regarding the other lines, we can say that there is low accession. Line of action 02, which concerns the intensification of strategic information on syphilis in maternal and child health services, had 07 related studies (36.84%), all with strategies for improving surveillance. This group is the one who have three studies that adhere to all lines of action, plus one carried out in Brazil.

Likewise, action line 03, which deals with improving laboratory network, quality management, and supply chain, also had one more study joining the three in South America, which made a total of 05 (26.31%). The fourth study activity was in the United States.

Finally, concerning transversal actions, which cover human rights, gender equality, and community, only the 06 (31.57%) studies from South America (21.05%), Asia (10.52%), and Europe (5.26%) mention engagement with the community and address the issue of human rights.

### Secondary analysis

Policies, when implemented and evaluated, serve as a guide and lesson learned so that countries can measure how much they have impacted on reducing the problem and what types of strategies should be adopted throughout their process to achieve the stipulated goal.

The present study analyzed secondarily and as a model to answer question 2 of this research, how the reviewed studies addressed or approached the implemented policies in evaluative terms.

Thus, nine surveys were observed that somehow evaluated the mentioned policy, or part of it was heeded. Table 3 summarizes this analysis:

**Table 3:** Distribution of studies regarding monitoring and evaluating syphilis policies.

**Authors**	**Year**	**Monitoring and evaluating?**
Taylor et al. (11)	2017	No
Leichliter et al. (12)	2017	No
Chemtob et al. (13)	2017	Epidemiologic and health policy data on STIs were analyzed from various sources
Rodrigues and Domingues (14)	2017	No
WHO (15)	2017	No
Akhtar and Rehman (16)	2018	No
Ong et al. (17)	2018	No
Leal et al. (18)	2018	Analyzed indicators of antenatal and labor and delivery care and maternal and infant health
Kroeger et al. (19)	2018	No
Andrade et al. (20)	2020	Chi-square tests were used to compare the proportions of adolescents and adults with a record of these procedures in the prenatal cards
Acharya et al. (21)	2020	Used data from the most recent nationally representative Nepal Health Facility Survey (NHFS) 2015
Liu et al. (22)	2020	No
Kimball et al. (23)	2020	No
Herrero et al. (24)	2020	No
Priamo et al. (25)	2020	No
Andrade et al. (26)	2020	Time series of Congenital Syphilis hospitalizations before and after the implementation of the “No Syphilis” Project in the Priority municipalities for the project and in other municipalities
WHO (27)	2020	Methods of pre-validation assessment included reviews of forms and registries used to record information at a facility level on ANC uptake, HIV and syphilis testing and treatment in pregnant women, HIV and syphilis case reporting in pregnant women and children, and reporting on infants and children exposed to these infections
Sykes et al. (28)	2021	Used surveillance data from January 1, 2017, through June 30, 2018, from three sources
Pinto et al. (29)	2021	Scrutinized seven data sources from different perspectives to assess a health campaign launched in Brazil named “Syphilis No!”. Developed a multidimensional analysis framework and implemented an information system to process the data from a time series perspective, and assessed the effects over time, both before and after the campaign. Analyzed data related to the campaign, including e-news, search engine activity, online courses, serological tests, medication distribution and case notification rates
PAHO (30)	2021	The model was calibrated to national data on syphilis prevalence, adult and congenital syphilis case notifications, risk behaviors, intervention coverage, test and condom procurement, and distribution volumes, and service delivery costs from routine surveillance, surveys, research studies, and program records
Zorilla et al. (31)	2021	Epidemiological data from Puerto Rico was used to document the elimination of MTCT and Syphilis. Data to calculate the indicators was obtained from the various divisions of the Puerto Rico Department of Health, including vital statistics, surveillance data, and programmatic outcomes
Du et al. (32)	2022	No
Tang et al. (33)	2022	Interrupted time series analysis was conducted to compare changes in slope and level of CS notification rate from 2005 to 2020 in Guangdong Province and its three regions with different economic developmental levels. The ARIMA model was established to predict the new CS case number of Guangdong Province in 2021

Source: Prepared by the authors (2022).

In Table 3, it is possible to observe that 11 studies carry out some action related to monitoring syphilis. The main elements presented were epidemiological and health policy data (13, 28, 31, 33); indicators of antenatal care, childbirth, and maternal and child health (18, 31, 33); proportions of adolescents and adults with records of procedures in prenatal cards (20); service information forms and records related to antenatal care, HIV and syphilis testing and treatment in pregnant women, HIV case reporting (27); epidemiological data and communication data (29).

Table 3 shows that six of the nine studies were carried out in South America: Leal et al. (18); Andrade et al. (20); Andrade et al. (26); PAHO (30); Pinto et al. (29); Zorilla et al. (31), who adhered to monitoring through some evaluative approach.

Leal et al. (18) presented an overview of public sector interventions and advances in women's and children's health in Brazil between 1990 and 2015. The authors reviewed public interventions, focusing on SUS and outcome indicators for reproductive, maternal, newborn, and child health (RMNCH). Also analyzing maternal data, Andrade et al. (20) assess adherence to the Brazilian recommendations for prenatal care. The authors analyzed data from the Adolescence and Motherhood Research (AMOR) Project and assessed adhesion to national recommendations as documented in the antenatal care cards of 39 adolescents (13–18 years) and 37 adults (23–28 years) from a low-income area in northeast Brazil.

Andrade et al. (26) tested the hypothesis that the intervention of the Project “Syphilis No!” of SUS in Brazil had influenced the decline in hospitalizations for CS in Brazilian municipalities as of May 2018. The authors compared the time series of hospitalizations for CS before and after implementing the project mentioned above in municipalities across the country. In the same vein and also in Brazil, Pinto et al. (29) presented an exploratory and descriptive analysis of data from communication campaigns implemented by “Syphilis No!” Project; the news indexed by Google about syphilis; of the number of tests and number of cases of syphilis in Brazil. The authors considered for the analysis three dimensions contemplated by the national policy of response to syphilis in Brazil: Communication, education, and epidemiological surveillance. They developed time series to evaluate the variables of interest over time.

The Pan American Health Organization (PAHO) presented an analytical report on strategies to eliminate syphilis in Peru, based on the systematization of epidemiological and programmatic data on syphilis, with the calibration of a model developed by researchers to project various scenarios that simulate the transmission of syphilis in adults. This model, widely used for HIV/AIDS, was used as a Pilot in Peru and prioritized analyzing the impact and cost-effectiveness of different STI control programs and interventions (30).

In Puerto Rico, the research implies the elimination of HIV and syphilis since 2007, which has been sustained, according to the data analyzed from 2005 to 2016 (31). Following the study conducted by Tang et al. (33), it is possible to verify in Guangdong Province in China, the reduction of CS from 2012 to 2020 by 94.65%.

## Discussion

The discussion focused on the research questions that this review aimed to answer.

1) How was the response to syphilis implemented in countries after the publication of the global strategies related to STIs and congenital syphilis proposed by the WHO in 2016?

Based on the scenario found in the results, it was possible to understand that 23 surveys reported some type of policy for coping with syphilis. America, Asia, Europe, and Africa presented studies at the continental level. Regarding the studies distribution by continent, the Americas region represents 65.21% of the publications, followed by Asia with 21.73%, Europe with 8.69%, and Africa with 4.37%. In 2021, the WHO published data from the previous year (7) on the increase in STIs, noting that the African continent has the highest incidence of syphilis, and America is in second place. This information is quite relevant, as it demonstrates a positive scenario in terms of publications carried out in the Americas region, which has advanced on the subject.

Following the studies reviewed, it is observed that there is still little integration between research on syphilis and the entire policy of eliminating this disease in the countries studied. In most of them, policies are partially described, making it difficult to correlate with WHO international documents.

The studies address elements of both international documents, limiting themselves to policy implementation strategies and focusing primarily on SIP and CS. Despite the relevance of this target audience for achieving the elimination of MTCT of syphilis, there was a lack of studies that consider other key populations, vulnerable populations, as well as mentioned, in a more relevant way, the active search for partners, which is part of the response to acquired syphilis, as recommended by the WHO (2016) (4) integrated with equity actions in access to universal health services.

As for the mention or adherence of articles and documents to all the strategies or lines of action of the 2016 WHO and 2017 PAHO/WHO frameworks, it was found that only 03 of the analyzed studies did so. Two of them were carried out in Brazil and one in Peru (26, 29, 30), which demonstrates that the Region of the Americas has been occupying a prominent place.

With regard to epidemiological surveillance, which is notably the main point for monitoring reported cases of syphilis, there are difficulties presented in the studies both for access and for the improvement of information, in addition to data processing, which makes it difficult to produce timely information to define the most up-to-date scenario on the situation of WHO signatory countries, with regard to the elimination of STIs, and CS, by 2030. For example, in Brazil, the National System for Notification of Diseases (SINAN), used to report syphilis cases in the SUS, is not integrated with the other technology platforms of the Ministry of Health (MH), particularly those of primary health care, an aspect that creates several gaps for the management of syphilis cases (34).

Another point that was not addressed is the equity of access to health services for STIs and syphilis. The studies analyzed show little transparency on governments regarding the operationalization of health policy in their territories. There is little clarity regarding the universal aspects and integrality of access to the health system and services, their contribution to reducing inequalities, and their consequent impact on STI rates. The lack of transparency makes it difficult to conduct public health policy to respond to syphilis epidemics and can be attributed, above all, to the lack of timely and more qualified management of cases and data generated by surveillance and information systems in countries.

The results found and discussed from the analyzed studies demonstrate that the financing and sustainability of policies are areas of concern. Financing is cited only for plans and projects in Brazil, Puerto Rico, and China (26, 29, 31–33). In Peru (30), however, no direct reference was found to a specific state project to fight syphilis, even though they described long-term programmatic actions in their analyses.

In the study by Andrade et al. (26), the time series of hospitalizations for CS before and after the implementation of “Syphilis No!” Project in Brazilian municipalities were compared. Significance was identified in the reduction of hospitalizations for CS that occurred after the implementation of the Project.

In terms of innovation for the future, the study by Pinto et al. (29) developed and used a platform called “Hermes” as a digital health solution based on computational intelligence, which allows health authorities to evaluate the conduct of any public health policy. The platform combines heterogeneous databases in scientific research, health education, health communication, health care at primary, secondary, and tertiary levels, and health surveillance data. This innovation allowed the development of analyzes and timely assessments during the duration of the intervention, which draws a lot of attention, given the importance of making timely decisions in health from a more integrated view of the actions and interventions developed in a given health system.

Thus, we proceeded with a verification of the context of the “Hermes” Platform application, and a second use was identified, which, being more recent, did not integrate this scoping review. In this second study, published in 2022 (35), the researchers expand the analysis of public health interventions to fight syphilis, focusing on the agenda of inter-federative articulations and actions that are part of the governance of SUS in Brazil. This aspect corresponded to yet another synergy between the analyzed data and increased the analysis on improving results of the response to syphilis. Studies carried out with syphilis data in Brazil demonstrate there is still an enormous potential for innovation in public health.

It is important to highlight that Brazil implemented an Interfederative Project entitled “Syphilis No!” in 2018, which was implemented in more than five thousand municipalities, having produced significant results when considering the cultural diversity and size of the country, not counting the intrinsic elements to the three inter-federative levels of the SUS and the insertion of the policy in the respective government plans. Recent studies have shown that a change in trend and righteousness is possible to eliminate this STI (35, 36).

2) Were the response to syphilis in these countries assessed and the methods used to assess these interventions?

The evaluation in terms of the health policy implemented is critical. It is possible to understand the future needs, investments, and improvements in the response capacity of a given country and its health system. From this, governments, researchers, and other organizations can base themselves on drawing up sustainable plans and strategies given the agreed goals. It is the thermometer in terms of what will be accomplished, lessons learned, and what can be replicated so that other countries achieve the elimination of STIs, such as syphilis.

Among the selected studies, 11 presented one or more attributes with evaluative methods. The highest percentage was from evaluations by statistical and computational methods, with two highlights found. One approach used multivariate analysis based on heterogeneous data sources in Brazil, concerning the variables of health surveillance, health communication, health education, research in the area of syphilis, and health care at all levels of care (29); and the other approach in Peru (30), carried out by the Pan American Health Organization (PAHO), using the SITE tool (Syphilis Interventions toward Elimination). Both were published in 2021.

The study by Pinto et al. (29) developed an exploratory analysis of syphilis using the following criteria: The actions of the syphilis communication campaign grouped and distributed over time; the population's interest in the topic of syphilis; offering courses and engaging the population; and indicators related to testing, treatment and the number of syphilis cases in Brazil. This corroborates with the scope of the strategies and their respective directions (who and where), how the intervention was carried out, the equitable delivery of health services, financing and sustainability, and, finally, innovation for the future.

To results achieved by Pinto et al. (29), it was possible to highlight that the activities of the search engine (Google) had a higher volume during the first week of the campaign in 2018 (between November 25 and December 7), which also showed a sustained growth trend until the end of 2019. In addition, data showed that 12 new online courses related to syphilis disease were available on the AVASUS Platform, to support efforts to promote lifelong learning, life for health professionals, teachers, and students. These courses reached more than 22,000 students between February 2019 and September 2020. Data from serological tests showed that the number of tests carried out in 2019 was 375.18% higher than in 2015, even accounting for population growth. Finally, starting in mid-2018, syphilis case notification rates followed a downward curve.

The tool applied in Peru (30), entitled SITE, was used in a pilot study between June and September 2020. The calibration of the model highlights the significant impact that Peru's HIV/STD program had on syphilis transmission between 2000 and 2019. The decline is particularly pronounced in sex workers and their clients and reflects the increase in condom use as well as the implementation of periodic examinations. In contrast, syphilis rates in MSM (men who have sex with men) and bisexual men remained high. Thus, the results were discussed in terms of impact on health and transmission, levels of service, and cost of alternative and combined packages of prevention, screening and treatment interventions.

It is essential to highlight the low number of studies dedicated to evaluating interventions. Regarding the studies found, they were presented in a heterogeneous way in terms of their analysis in terms of data and objectives. Thus, the present study was restricted to understanding what was accomplished and how close the countries are to achieving the goals set by the WHO 2016 (4).

It was possible to verify that countries such as Cuba, Thailand, Belarus, Armenia, Moldova (11), and Puerto Rico (31) reported the elimination of MTCT of syphilis. The actions and policies carried out were described based on the strategies for the elimination of SIP and CS, based on universal health access, syphilis prevention efforts, prenatal care, testing, treatment, and monitoring of cases. The declaration of elimination was first made by Cuba in 2015, and in the remaining countries (Thailand, Belarus, Armenia, and Moldova) in 2016 (11). After the period, no new studies with more recent data were found. In Puerto Rico (31), research reports that since 2007 elimination has been maintained, but it is important to emphasize that data were also analyzed up to 2016.

More recently, research carried out in Brazil, after the period of search of this article, developed through computational methods (36) the evaluation of syphilis response which applied automated text mining methods to understand how the interventions of the field in Brazil impacted the reduction of syphilis in the municipalities designated as a priority. In this way, it was possible to verify a 15.75% reduction in the reduction of CS through research and intervention actions carried out in the period from 2018 to 2019, which characterizes an important change in the trend for new CS cases in the last few years 10 years in Brazil (36), which used to be an increase.

Finally, it is important to mention that the main finding of this research is that there is still a lack of more recent updates on how countries have carried out the policy response to syphilis globally and whether they are close to achieving the SDGs of Agenda 2030 and the target set by the WHO (4). As can be seen, research is highly relevant because it can contribute to national and international decision-making and policymaking, particularly through reported best practices, lessons learned, adaptations, and innovations used by countries.

## Concluding remarks

Based on the studies analyzed, it is possible to conclude that since the launch of the WHO Policy in 2016, few studies have been carried out focusing on the description and evaluation of public policies in response to syphilis. Most of them are limited to mentioning the implementation of guidelines related to the WHO Strategy 02 (2016), and there is still a lack of clarity about how equity is promoted, investment in policy, and possible innovations for the future. In terms of eliminating CS, the main focus of the studies was related to Line of Action 01 of the PAHO Framework, with emphasis on issues related to health services in prenatal care, testing, and treatment, and with a lot of limitations regarding discussions related to partner follow-up and the occurrence of reinfections.

The highlight for Brazil, which implemented a national project that induced universal and well-targeted actions for a response at municipal level to change the trend of the epidemic, bringing analytical results focused on the response to CS, as well as for Peru and China, which invested and participated in an analysis of the national scenario capable of promoting changes in the course of its policy based on the results found in specific populations and on its experience in response to HIV/AIDS. Both countries promoted evaluative-type studies.

In general terms, it is worth mentioning the lack of data for monitoring and surveillance, which is also a gap and makes it difficult to assess how actions can reduce and impact the rates of acquired syphilis, SIP, and CS around the world. Therefore, there is an urgent need for studies that align actions, processes, and adhesion to the recommendations and the proposed Framework, to bring directions for future research and make science able to contribute to the decision-making process as well as to health policies to be international, national, regional and local level. Among the studies found, six countries (11, 31) reported the elimination of MTCT of syphilis, but the most recent data are from 2016. Furthermore, it is essential to mention that no countries were found that have eliminated syphilis in its entirety.

## Data availability statement

The datasets presented in this study can be found in online repositories. The names of the repository/repositories and accession number(s) can be found in the article/supplementary material.

## Author contributions

MA: Study design, data and article collection, analysis and interpretation of results and drafting of the manuscript. AC and AC-O: Guidance and revision of the manuscript. DB, TL, and RV: Analysis and interpretation of the results and revision of the manuscript. DS: Study design, data and article collection. All authors contributed to manuscript revision, read, and approved the submitted version.

## Funding

This article is funded through the Decentralized Execution Term no. 54/2017 in cooperation with the Brazilian Ministries of Health and the Federal University of Rio Grande do Norte.

## Conflict of interest

The authors declare that the research was conducted in the absence of any commercial or financial relationships that could be construed as a potential conflict of interest.

## Publisher's note

All claims expressed in this article are solely those of the authors and do not necessarily represent those of their affiliated organizations, or those of the publisher, the editors and the reviewers. Any product that may be evaluated in this article, or claim that may be made by its manufacturer, is not guaranteed or endorsed by the publisher.

## Abbreviations

CS: congenital syphilis
HIV: human immunodeficiency virus
MTCT: mother-to-child transmission
PAHO: Pan American Health Organization
PCC: Population, Concept and Context
SIP: syphilis in pregnancy
STI: sexually transmitted infection
*T. pallidum*: *Treponema pallidum* subsp *Pallidum*
UN: United Nations
WHO: World Health Organization.
